# Sphingosine-1-Phosphate Alleviates Irradiation Induced Salivary Gland Hypofunction through Preserving Endothelial Cells and Resident Macrophages

**DOI:** 10.3390/antiox11102050

**Published:** 2022-10-18

**Authors:** Tao Yang, Qingguo Zhao, Meijun Hu, Simin Pan, Linying Zhang, Ruoxi Zhu, Bowen Zhou, Xuanhe Feng, Zhenhua Gao, Zhao Zhu, Yu Zhang, Liang Hu, Fei Liu, Zhaochen Shan

**Affiliations:** 1 Outpatient Department of Oral and Maxillofacial Surgery, School of Stomatology, Capital Medical University, Beijing 100050, China; 2 Department of Cell Biology and Genetics, School of Medicine, Texas A&M University Health Science Center, College Station, TX 77843, USA; 3 Department of Statistics, Texas A&M University, College Station, TX 77843, USA; 4 Salivary Gland Disease Center and Beijing Key Laboratory of Tooth Regeneration and Function Reconstruction, Beijing Laboratory of Oral Health, Capital Medical University School of Stomatology, Beijing 100050, China

**Keywords:** sphingosine-1-phosphate signaling, salivary glands, irradiation, head and neck cancers, endothelial cells, resident macrophages

## Abstract

Radiotherapy for head-and-neck cancers frequently causes long-term hypofunction of salivary glands that severely compromises quality of life and is difficult to treat. Here, we studied effects and mechanisms of Sphingosine-1-phosphate (S1P), a versatile signaling sphingolipid, in preventing irreversible dry mouth caused by radiotherapy. Mouse submandibular glands (SMGs) were irradiated with or without intra-SMG S1P pretreatment. The saliva flow rate was measured following pilocarpine stimulation. The expression of genes related to S1P signaling and radiation damage was examined by flow cytometry, immunohistochemistry, quantitative RT-PCR, Western blotting, and/or single-cell RNA-sequencing. S1P pretreatment ameliorated irradiation-induced salivary dysfunction in mice through a decrease in irradiation-induced oxidative stress and consequent apoptosis and cellular senescence, which is related to the enhancement of Nrf2-regulated anti-oxidative response. In mouse SMGs, endothelial cells and resident macrophages are the major cells capable of producing S1P and expressing the pro-regenerative S1P receptor S1pr1. Both mouse SMGs and human endothelial cells are protected from irradiation damage by S1P pretreatment, likely through the S1pr1/Akt/eNOS axis. Moreover, intra-SMG-injected S1P did not affect the growth and radiosensitivity of head-and-neck cancer in a mouse model. These data indicate that S1P signaling pathway is a promising target for alleviating irradiation-induced salivary gland hypofunction.

## 1. Introduction

Head and neck cancers (HNCs) are the 7th most common cancers worldwide in 2020 with an estimated 931,931 new cases and 387,117 new deaths [1]. Radiation therapy is a first-line HNC treatment but frequently causes irreversible hypofunction of the salivary glands [2]. Even with the progress of intensity-modulated radiation therapy, the incidence and severity of irradiation (IR)-induced dry mouth remain high [3]. IR-induced dry mouth leads to difficulties in sleep, speech, chewing, and swallowing and increases the risk of dental caries, which severely compromises the patients’ quality of life [4]. Current treatments, such as artificial saliva and saliva secretion stimulators, only partially and temporarily alleviate these symptoms. Therefore, effective therapies for IR injury of salivary glands are critically needed.

In both mouse and miniature pig models, microvascular endothelial cells in the salivary glands were rapidly (from 4 h after IR) and persistently damaged by IR [5,6]. Our recent findings indicated that salivary gland resident macrophages also decreased rapidly after IR and were essential for preserving salivary gland function through homeostatic paracrine/juxtacrine interactions with endothelial cells and epithelial progenitors [7,8]. Therefore, approaches targeting these two types of cells are promising for the preservation or restoration of saliva secretion after IR [5,6,7,8].

Sphingosine-1-phosphate (S1P) is a bioactive sphingolipid produced by ceramide metabolism and functions, mainly in the secreted form through cell surface S1P receptors [9]. S1P receptors (S1pr1-5) are G-protein-coupled receptors, and S1P/S1pr interactions activate intracellular signaling molecules such as protein kinase B (Akt) to regulate various pathophysiological processes including cell proliferation, migration, apoptosis, and angiogenesis [9]. S1P is mainly produced and secreted by endothelial cells, erythrocytes, and platelets, while the major cells responsive to extracellular S1P in most organs are endothelial cells and macrophages [9].

We previously showed that, in a miniature pig model, intra-parotid administration of S1P alleviated IR-induced parotid injury [10], but the underlying mechanisms remain unclear due to the limited tools for studying pig molecules. Therefore, we switched to a mouse model with sufficient established research tools for mechanism study and focused on the submandibular glands (SMGs) that are the major source of both resting and stimulated saliva [11]. Here, we report that intra-SMG-injected S1P alleviates the deleterious effects of IR on salivary gland function, which is achieved by decreasing oxidative stress and consequent DNA damage, apoptosis, and cellular senescence, mainly in endothelial cells and resident macrophages, likely through the activation of the S1pr1/Akt pathway and the enhancement of Nrf2-regulated antioxidative responses.

## 2. Materials and Methods

### 2.1. Animal Experiments

C57BL/6 mice for single-cell RNA sequencing and flow cytometry were purchased from the Jackson Laboratory (Maine, USA). Other C57BL/6 mice and nude mice were purchased from SPF Biotechnology (Beijing, China). S1P (Cayman Chemicals, Ann Arbor, MI, USA) was first dissolved in a PET vehicle (5% polyethylene glycol 400, 2.5% ethanol, and 0.8% Tween-80 diluted in PBS) [10] at a concentration of 1 mg/mL and then diluted with PBS before use. C57BL/6 mice were randomly grouped to the following treatments: non-treated (NT), intra-SMG PET injection (vehicle), intra-SMG S1P injection (S1P), PET injection followed by 15 Gy irradiation (IR), and S1P injection followed by IR (IR + S1P). S1P was injected percutaneously into mouse SMGs 30 min prior to IR at the dose of 0.05, 0.1, or 0.2 mg/kg body weight. PET was similarly injected at an equivalent dose to that for 0.2 mg/kg S1P. The pilocarpine-stimulated salivary flow rate was measured weekly from one week before treatment for 5 weeks and then at day 60 and 90 after treatment from mice anesthetized intraperitoneally with ketamine (100 mg/kg) and xylazine (10 mg/kg). Blood was collected from tail vein for detecting plasma S1P concentration. Nude mice were injected subcutaneously with CAL27 cells in their flanks, as previously reported [12]. Tumor volumes were monitored every three days, and mice were randomly grouped when tumor sizes reached approximately 0.3 cm^3^. Tumor-carrying mice were treated with intra-SMG injection of 0.2 mg/kg S1P as mentioned above, 15 Gy single-dose irradiation at the tumor site, as we previously reported [13], or both. These mice were observed for 15 days after the above treatment and then euthanized.

### 2.2. Cell Culture

Human umbilical vascular endothelial cells (HUVECs) and VascuLife medium were purchased from Lifeline Cell Technology (FC-0003 and 08837, San Diego, CA, USA) and cultured at atmospheric oxygen levels (20% O_2_; 5% CO_2_). Passage 3 (P3) HUVECs were used in all experiments. Human CAL27 head and neck cancer cells were purchased from ATCC (CRL-2095) and cultured as previously described [12]. S1P was dissolved in a PET vehicle to 0.1, 0.5, or 1 μM and added to the cell culture 30 min before IR. A selective S1pr1 antagonist, W146 (Tocris Bioscience, Bristol, UK), was dissolved in 10% DMSO+ 25% Tween 20 as a 20 mM stock and added 1 h before IR into culture medium with a final concentration of 10 μM. Akt inhibitor, MK2206 (MCE, HY-10358, Monmouth Junction, NI, USA), was dissolved in PBS.

### 2.3. Irradiation

Animals were anesthetized with ketamine (100 mg/kg) and xylazine (10 mg/kg) and then received a 15 Gy single dose of IR at the submandibular gland area (Supplementary Figure S1). The IR was performed with a photon energy of 6 mV at 3 Gy/min using an Elekta Synergy accelerator (Trilogy Varian, Palo Alto, CA, USA). HUVECs received 10 Gy single-dose IR at 1.48 Gy/min using the same accelerator.

### 2.4. Biochemical Test

SOD activity in the submandibular gland homogenate was detected using the Total Superoxide Dismutase Assay Kit (S0101, Beyotime, Shanghai, China) according to the manufacturer’s instructions.

### 2.5. Immunofluorescence (IF) and Immunohistochemistry (IHC) Staining

SMG sections and HUVECs were fixed in 4% paraformaldehyde for 30 min, rinsed using PBS 3 times, and then blocked with 10% goat serum for 1 h at room temperature. These samples were incubated overnight at 4 °C with following primary antibodies: anti-p-H2ax (1:200, 80312, CST, Danvers, MA, USA), anti-Nrf2 (1:50, 16396, Proteintech, Rosemont, IL, USA), anti-S1pr1 (1:500, PA11040, ThermoFisher Scientific, Waltham, MA, USA), anti-Cd31 (1:50, ab281583, Abcam, Waltham, MA, USA), and anti-F4/80 (1:100, bs7058R, Bioss, Beijing, China). After washing, samples were incubated for 1 h at room temperature with corresponding secondary antibodies: Alexa Fluor^®^ 594-conjugated goat anti-rabbit IgG (1:500, AS039, ABclonal, Wuhan, China), Alexa Fluor^®^ 488-conjugated goat anti-mouse IgG (1:500, ABclonal AS037), Alexa Fluor^®^ 488-conjugated goat anti-rabbit IgG (1:500, Abcam ab150077), and Alexa Fluor^®^ 594-conjugated goat anti-mouse IgG (1:500, Abcam ab150115). After washing, samples were counterstained with 4′,6-diamidino-2-phenylindole (DAPI) (F6057, Sigma, St. Louis, MO, USA). For IHC, the secondary antibody used was labeled with HRP (1:10,000, Abcam ab205718), DAB was used as the chromogen, and the sections were counterstained with hematoxylin. All IF and IHC images were captured using a microscope (BX61, Olympus, Tokyo, Japan). Six representative fields from three independent samples in each group were quantified to calculate the positively stained areas or positive cells.

### 2.6. Detection of Reactive Oxygen Species (ROS)

Intracellular ROS levels in HUVECs were detected using an ROS assay kit (S0033, Beyotime, Shanghai, China) according to the manufacturer’s instructions. The stained ROS signals were then photographed using a fluorescent microscope (OLYPUS BX 61, Tokyo, Japan).

### 2.7. Western Blot

The submandibular glands and HUVECs were ground on ice, and proteins were harvested using lysis buffer (C1055, Applygen, Beijing, China) containing a protease inhibitor cocktail (P8340, Sigma, Saint Louis, MO, USA) and phosphatase inhibitors (P1261, Applygen, Beijing, China). Protein concentration was determined using the Bradford method (Bio-Rad Laboratories, Hercules, CA, USA). Equal amounts of protein (25 μg) were separated by protein electrophoresis and transferred to PVDF membranes. PVDF membranes were incubated overnight with primary antibodies including Aqp5 (0.1 μg/mL, ab78486, Abcam, Waltham, MA, USA), Bax (1:1000, Abcam ab32503), Bcl2 (1:2000, Abcam ab182858), p-H2ax (1:1000, CST 80312, Danvers, MA, USA), cleaved Caspase3 (1:1000, CST 9664P), p53 (1:1000, 10442-1-AP, Proteintech, Rosemont, IL, USA), Nox4 (1:1000, Proteintech 14347), Nrf2 (1:1000, Proteintech 16396), Akt (1:1000, Proteintech 10176-2), p-Akt (1:2000, CST 4060T), Sod2 (1:1000, Abcam ab137037) eNOS (1:500, Abcam ab76198), p-eNOS (1:1000, CST 9570), and β-actin (1:50,000, Abclone AC026). After washing, the membranes were incubated with secondary antibodies (Goat anti-rabbit or anti-mouse IgG, 1:10,000, Abcam ab97051 and ab97040) for 1 h at room temperature. The proteins examined were visualized with Clarity Western ECL Substrate (Bio-Rad 170-5060, Hercules, CA, USA). Protein levels were analyzed as band intensities with the Image J software, normalized with the loading control β-actin first, and then shown as relative levels compared to normalized levels of corresponding proteins in nontreated group or vehicle group.

### 2.8. Reverse Transcription-Quantitative Polymerase Chain Reaction (RT-qPCR)

RNAs were extracted from SMGs using the RNeasy Mini Kit (Qiagen, Redwood City, CA, USA) and reverse transcribed using the High-Capacity cDNA Reverse Transcription Kit (Applied Biosystems, Waltham, MA, USA). The primer sequences were retrieved from Primerbank (http://pga.mgh.harvard.edu/primerbank, accessed on 11 October 2021) and synthesized by Invitrogen (Waltham, MA, USA). qPCR was performed using SYBR Green Master Mix (Bio-Rad, Hercules, CA, USA) on a 7900HT Fast Real-Time PCR System (Applied Biosystems, Waltham, MA, USA). mRNA levels were normalized with the reference gene *Gapdh* first, and then shown as relative levels compared to normalized levels of corresponding genes in nontreated group unless stated otherwise.

### 2.9. Assays on Apoptosis and Cellular Senescence

Fixed frozen sections (5 μm) were prepared from the SMGs. Terminal deoxynucleotidyl transferase biotin-dUTP nick end labeling (TUNEL) and senescence-associated β-galactosidase (SA-βgal) staining were performed using the One Step TUNEL Apoptosis Assay Kit (C1090, Beyotime, Shanghai, China) and SA-β-gal staining kit (C0602, Beyotime, Shanghai, China) according to the manufacturer’s instructions. TUNEL-positive cells and SA-β-gal-positive areas were calculated using the ImageJ software (Version 1.53t 24, NIH, Bethesda, MD, USA). Six representative fields from three independent samples in each group were quantified to calculate the positively stained areas or positive cells.

### 2.10. Single-Cell RNA Sequencing (scRNA-Seq) and Bioinformatics Analyses

Single-cell suspensions were isolated from mouse SMG as previously reported [7]. Briefly, SMGs were minced and digested for 1 h with RPMI 1640 medium containing 1 mg/mL collagenase IV, 5 mM CaCl_2_, 50 mg DNase I, and 8% fetal bovine serum with continuous shaking at room temperature. scRNA-seq libraries were generated in TAMU Genomics Core using the 10× Chromium platform and the version 3 Chromium Single Cell 3′ reagent kit following the manufacturer’s instructions (10× Genomics, Pleasanton, CA, USA). Samples were pooled in equimolar concentrations and sequenced on a single lane of an S4 2 × 150 PE flow cell on an Illumina NovaSeq 6000 (Illumina, San Diego, CA, USA). Bioinformatics analyses were performed by researchers blinded to the treatments using the methods detailed in our recent paper [7]. The uniform approximation and projection method (UMAP) was used to visualize the clusters. The normalized data are shown as feature plots. Approximately 18,000 cells were sequenced at a depth of 1420 median genes per cell. The heatmap of S1P-related genes in major types of SMG cells was generated with globally distinguishing fold-change data exported from the Loupe Browser (Version 6.2.0, 10× Genomics, Pleasanton, CA, USA).

### 2.11. Flow Cytometry

Single cells from mouse SMGs were generated as mentioned above. These cells were stained with fluorescent-labeled antibodies against F4/80 (BD Pharmingen 123116, 123107, San Diego, CA, USA), Cd31 (BioLegend 102410, San Diego, CA, USA), S1pr1 (R&D Systems FAB7089P, Minneapolis, MN, USA), S1pr2 (Biorbyt orb319083-FITC, St Louis, MO, USA), S1pr3 (Bioss Bs-7541R-PE, Woburn, MA, USA), or the corresponding isotype controls (BioLegend 400608, 400511, 400612, 400207, 1:100, San Diego, CA, USA). Dead cells were excluded using a LIVE/DEAD Fixable Aqua Dead Cell Stain Kit (Invitrogen, Waltham, MA, USA). The stained cells were analyzed on an FC500 flow cytometer (Beckman Coulter, Brea, CA, USA). Data were analyzed using the FlowJo software (Version 10.8.1, FlowJo, Ashland, OR, USA).

### 2.12. Statistical Analysis

All data were analyzed with Prism 9 software (Version 9.4.1, GraphPad, San Diego, CA, USA) using One-way ANOVA with Tukey’s multiple-comparison test. Charts were generated using Prism 9. Statistical significance was set at *p* < 0.05.

## 3. Results

### 3.1. Locally Injected S1P Alleviates Irradiation-Induced Salivary Gland Hypofunction in Mice

**Figure 1:**
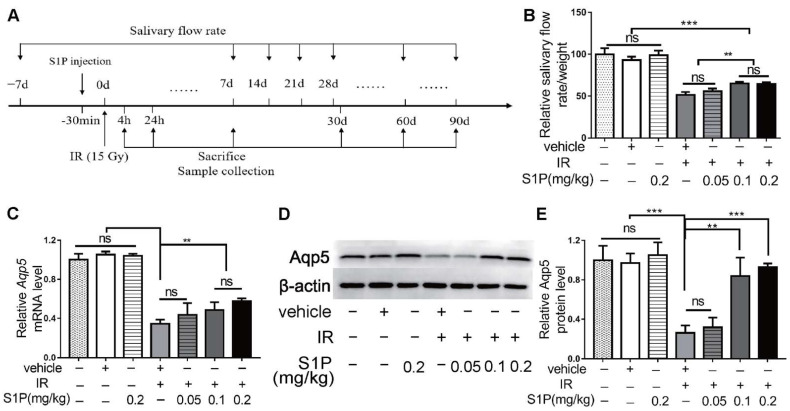
**Locally injected S1P prevented irradiation damages on salivary gland function.** (**A**) The experimental design. (**B**) The pilocarpine-stimulated whole salivary flow rate was measured 7 days before and 7, 14, 28, 60 and 90 days after IR (N = 6). (**C**–**E**) RT-qPCR and Western blot analysis of Aqp5 level in SMGs collected at 90 days after IR (N = 3). Data are expressed as mean ± SD, **: *p* < 0.01, ***: *p* < 0.001, ns: not significant. Quantitative data were analyzed using one-way ANOVA with Tukey’s multiple comparisons.

We previously reported that S1P administered into the parotid gland by retrograde ductal instillation alleviates irradiation (IR)-induced parotid injury in a miniature pig model, but the underlying mechanisms are largely unclear [10]. The mechanisms of IR-induced dry mouth and corresponding treatments have mostly been studied in mouse models, while submandibular glands (SMGs) produce approximately 2/3 of the total resting saliva and 40% of the total stimulated saliva [11]. Therefore, we used the mouse SMG model to determine the effects and mechanisms of intra-SMG-injected S1P on IR-induced salivary gland hypofunction. In brief, we percutaneously injected 0.05, 0.1, or 0.2 mg/kg S1P into SMGs of adult C57BL/6 mice and then radiated mice with or without S1P pretreatment 30 min later at the SMG region (Supplementary Figures S1 and S2). The doses of S1P used in our mouse study was based on our related study in mini-pigs (0.02 mg/Kg) [10] and the dose conversion ratio around 11 between these two species [14]. The pilocarpine-stimulated whole saliva flow rate in all mice was measured at 7 days before and 7, 14, 28, 60 and 90 days after S1P and/or IR treatment and normalized with body weight, and SMGs were collected on days 1, 7, and 90 after these treatments (Figure 1A and Figure S3). The normalized saliva flow rates at all time points were greatly reduced in the IR group compared with the nontreated (NT) group, as expected, while the pretreatment of S1P at 0.1 or 0.2 but not 0.05 mg/kg significantly improved the saliva flow rate compared with the IR group at 28, 60 and 90 days after IR (Figure 1B). Consistently, the expression of Aquaporin5 (Aqp5), a marker of saliva-producing acinar cells, was significantly decreased by IR, but preserved by 0.1 or 0.2, but not 0.05 mg/kg, S1P pretreatment at both the mRNA and protein levels at day 90 (Figure 1C–E). These data indicate that locally administered S1P alleviates IR damage in mouse SMGs. Since no significant differences in the above indexes were found between 0.1 and 0.2 mg/kg S1P groups, and the polyethylene glycol 400 in S1P vehicle should be minimized to avoid potential negative effects [15], we used 0.1 mg/kg S1P for all following experiments.

### 3.2. S1P Decreases Irradiation-Induced Apoptosis and Cellular Senescence in Salivary Glands

**Figure 2:**
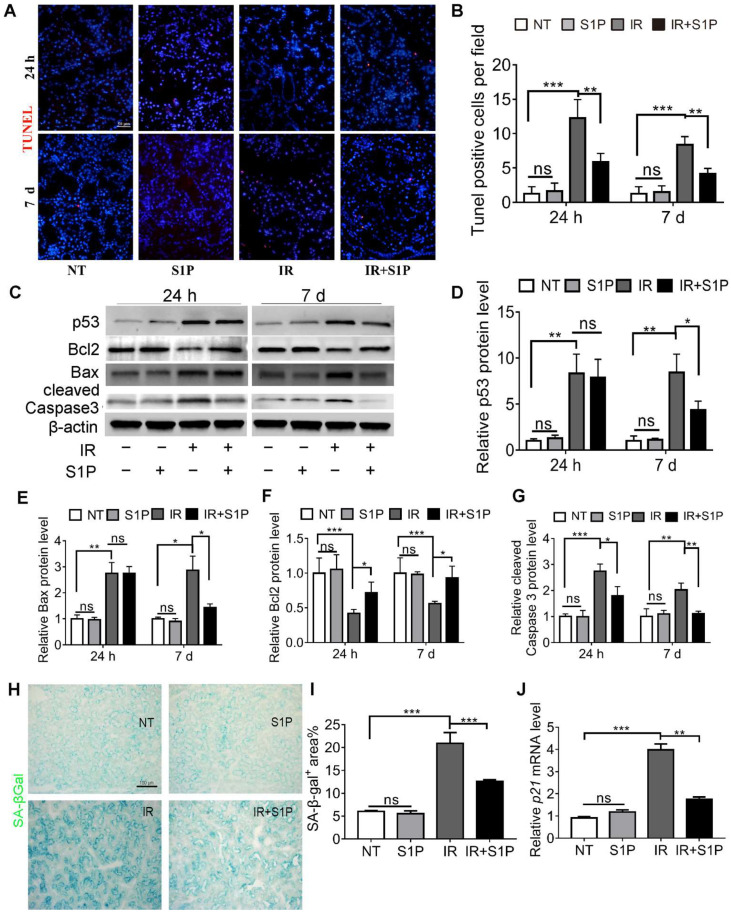
**S1P decreased apoptosis and cellular senescence in radiated salivary glands.** (**A**,**B**) TUNEL staining of SMG frozen sections collected 24 h or 7 days after IR or S1P treatment. Magnification: ×400. (**C**–**G**) Western blot analyses of Bax, Bcl 2, p53, and cleaved Caspase 3 proteins. (**H**,**I**) Senescence-associated β-galactosidase (SA-β-Gal) staining and semiquantitative analysis of SA-β-Gal-positive areas. (**J**) RT-qPCR analysis of mRNA levels of *p21*. Data are shown as mean ± SD, N = 3, *: *p* < 0.05, **: *p* < 0.01, ***: *p* < 0.001, ns: not significant. Quantitative data were analyzed using one-way ANOVA with Tukey’s multiple comparisons.

IR-induced dry mouth is related to apoptosis and the senescence of salivary gland cells in both mouse and pig models [16,17,18,19,20]. TUNEL staining of SMGs collected 24 h and 7 days after IR indicated that the number of apoptotic cells significantly increased after IR, as expected, which was significantly repressed by S1P pretreatment (Figure 2A,B and Figure S4). Consistently, at both 24 h and 7 d after IR, pro-apoptotic proteins p53, Bax, and cleaved caspase 3 were upregulated in the IR group and decreased in the IR + S1P group, while the anti-apoptotic protein Bcl2 was downregulated by IR and increased in the IR + S1P group (Figure 2C–G). Senescence-associated β-galactosidase (SA-β-Gal) staining indicated that the cellular senescence induced by IR was significantly inhibited by S1P pretreatment 7 days after IR (Figure 2H,I). Consistently, the expression of the senescent marker p21 was significantly increased after IR but decreased by S1P on day 7 after IR (Figure 2J). In all the above assays, S1P treatment alone did not significantly affect any of the measured indexes. Taken together, these data suggest that S1P alleviates IR damage in mouse SMGs by decreasing IR-induced apoptosis and cellular senescence.

### 3.3. S1P Ameliorates IR-Caused Oxidative Stress and DNA Damage by Upregulating Nrf2-Related Antioxidant Responses

**Figure 3:**
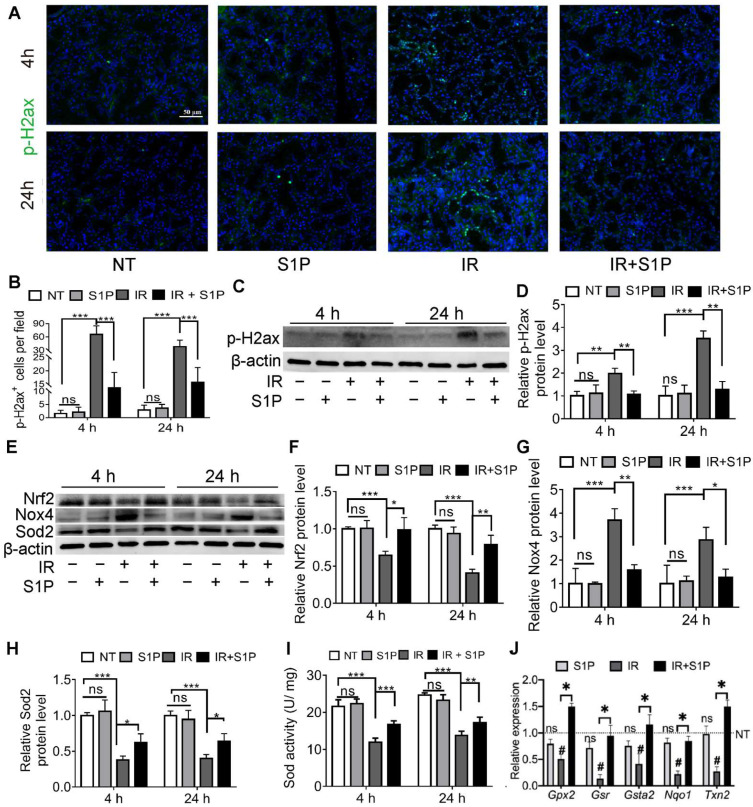
**S1P decreased oxidative stress and DNA damage in radiated salivary glands.** (**A**–**D**) Levels of p-H2ax in SMGs collected at 4 or 24 h after IR were examined with immunofluorescence staining and Western blot analyses. Magnification: ×400. (**E**–**H**) Levels of Sod2, Nox4 and Nrf2 proteins in SMGs were examined with Western blot. (**I**) Sod activities in SMG homogenate were examined with a commercial kit. (**J**) mRNA levels of Nrf2 target genes in SMGs collected at 24 h after IR were examined with RT-qPCR. Data are shown as mean ± SD, N = 3, ns: not significant vs. NT, #: *p* < 0.05 vs. NT, *: *p* < 0.05, **: *p* < 0.01, ***: *p* < 0.001. Quantitative data were analyzed using one-way ANOVA with Tukey’s multiple comparisons.

Cellular senescence in irradiated salivary glands is caused by oxidative stress and consequent DNA double-strand breaks (DSB) [18,19]. As indicated by immunofluorescence staining and Western blotting, the level of phospho-H2A.X variant histone (p-H2ax), a DSB marker, was remarkably increased in SMGs 4 and 24 h after IR, as expected, and was significantly repressed by S1P pretreatment (Figure 3A–D and Figure S5). Oxidative stress caused by IR is related to an increase in NADPH oxidase 4 (Nox4) in endothelial cells [21] and a decrease in superoxide dismutase 2 (Sod2) in mouse SMGs [22]. The expression of Sods and many other antioxidant enzymes is enhanced by nuclear factor erythroid 2-related factor 2 (Nrf2/Nfe2l2) in various mammalian tissues, including salivary glands [23,24], whereas Nrf2 protein levels are downregulated by IR [25] but upregulated by S1P signaling in cultured cells from several other organs [26,27]. As indicated by Western blot analyses of SMG homogenate at 4 and 24 h after S1P or IR treatment, IR decreased the expression of Sod2 and Nrf2 but increased Nox4 expression as expected, while S1P treatment alone did not significantly affect the expression of these three proteins in non-irradiated SMGs, but significantly reversed the effects of IR on these proteins (Figure 3E–H). Similar changes in the total SOD activity were found in the SMG homogenate using a commercial kit (Figure 3I). Consistently, the mRNA levels of multiple Nrf2-regulated anti-oxidative genes, including *Gpx2*, *Gsr*, *Gsta2*, *Nqo1*, and *Txn2* [28,29], in SMGs collected 24 h after IR were significantly decreased by IR but restored by S1P treatment before IR (Figure 3J). Taken together, these data indicate that the radioprotective effects of S1P are related to the upregulation of Nrf2-related antioxidant responses and the consequent decrease in oxidative stress and DNA damage.

### 3.4. Genes Required for S1P Production, Secretion, and Pro-Regenerative Signaling Are Mainly Expressed in Salivary Gland Endothelial Cells and Resident Macrophages

**Figure 4:**
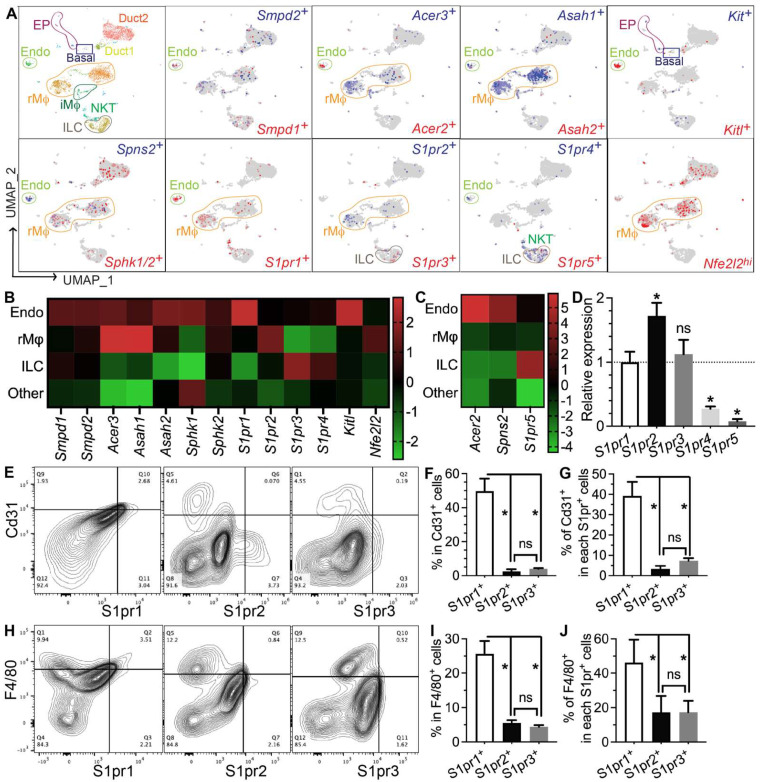
**Expression patterns of S1P metabolic genes and receptors in salivary glands.** (**A**–**C**) Expression pattern and relative levels of genes essential for S1P production and secretion, S1P receptors, and potential target genes of S1P signaling were shown in the feature plots (**A**) and heatmaps (**B**,**C**) of scRNA-seq data of nontreated SMGs. Endo: endothelial and peri-endothelial cells; rMφ: resident macrophages; iMφ: infiltrating macrophages; Epi: epithelial cells; ILC: innate lymphoid cells; NKT: natural killer T cells. The color scales in (**B**,**C**) are log2 fold changes vs. all other clusters. (**D**) mRNA levels of S1P receptors in nontreated SMGs were examined with RT-qPCR and shown as relative to *S1pr1*. N = 3. *: *p* < 0.05 vs. *S1pr1*; ns: not significant vs. *S1pr1*. (**E**–**J**) Expression patterns of major S1P receptors in SMG endothelial cells and macrophages were confirmed with flow cytometry. N = 3. *: *p* < 0.05, ns: not significant. Quantitative data were analyzed using one-way ANOVA with Tukey’s multiple comparisons.

To determine which types of SMG cells produce S1P and/or respond to S1P signaling, we performed single-cell RNA-sequencing (scRNA-seq) of SMGs from non-treated adult C57BL/6 mice and analyzed data as we recently reported [7]. Sphingosine is produced at the plasma membrane from ceramides by ceramidases (Acer1/2/3 and Asah1/2) and then phosphorylated by sphingosine kinases (Sphk1/2) to form S1P, while the exportation of S1P is mediated by spinster 2 (Spns2) from endothelial cells and ABC transporters from red blood cells [30]. Ceramide levels increase immediately after IR through the hydrolysis of sphingomyelin in cell membranes by the acid and neutral sphingomyelinases Smpd1 and Smpd2, which leads to apoptosis [31,32,33]. In our scRNA-seq data, most cells in the “Endo” cluster are *Pecam1^+^* endothelial cells and about 20% of them are peri-endothelial cells (*Acta2^+^* vascular smooth muscle cells, *Pcp4l1^hi^* pericytes, and *Pecam1^−^Cd34^+^* progenitors, Supplementary Figure S6A–E,Q), as reported in various other tissues [34,35,36]. *Smpd1* and *Smpd2* are most abundant in the Endo cluster, *Sphk1* and *Sphk2* are widely expressed in most types of cells, while all detectable ceramidases (*Acer2/3 and Asah1/2*) and the S1P transporters *Spns2* are enriched in the Endo cluster and resident macrophages (rMφs), suggesting that they are the main sources of extracellular S1P in mouse SMGs (Figure 4A–C). Notably, among the five S1P receptors, S1pr1 and S1pr5 signaling showed anti-inflammatory, vascular protective, and pro-regenerative effects [37,38,39], whereas S1pr2, S1pr3, and S1pr4 signaling pathways are reported to be pro-inflammatory and pro-fibrotic [40,41,42]. S1pr1 mRNA was enriched in the Endo cluster and present in multiple types of cells, including resident macrophages; S1pr2 mRNA was present in macrophages and ductal cells, while S1pr3/4/5 mRNAs were enriched in lymphocytes such as innate lymphoid cells (ILCs) and NKT cells (Figure 4A–C). In the Endo cluster, >93% *S1pr1^+^* cells are *Pecam1^+^* endothelial cells, while <7% *S1pr1^+^* cells are either *Pecam1^−^Cd34^+^* progenitors or *Pcp4l1^hi^* pericytes but not *Acta2^+^* smooth muscle cells (Supplementary Figure S6F,G,R). Among all other S1pr genes, only *S1pr4* was detected in the Endo cluster in a few cells (<5%), mostly in *Pecam1^+^* endothelial cells and occasionally in *Pcp4l1^hi^* pericytes (Supplementary Figure S6K–P,S). The expression of *Nrf2* (*Nfe2l2*), an S1P target gene essential for its radioprotective effect, is also enriched in rMφs (Figure 4A–C). SMG endothelial cells, another major S1P-responsive cell, are highly expression growth factors essential for the homeostasis of other cells, such as Csf1 for rMφs [7] and *Kitl* (stem cell factor) for *Kit^+^* epithelial progenitors (Figure 4A–C). Since most F4/80^+^ cells in SMGs are Ly6c^low^ resident macrophages (~90%) [7], we used F4/80 as a surrogate marker for SMG rMφs in flow cytometry assays. The expression patterns of the above genes were validated by RT-qPCR assays on CD31^+^, F4/80^+^, and CD31^−^F4/80^−^ (double negative) cells isolated from nontreated SMGs with FACS (Supplementary Figure S7). The RT-qPCR assay on mRNA from whole SMGs indicated that *S1pr1*, *S1pr2*, and *S1pr*3 are expressed at much higher levels than *S1pr4* and *S1pr5* (Figure 4D). Consistently, as indicated by flow cytometry assays, most S1pr1^+^ cells in SMGs express either the endothelial marker CD31 (~40%) or F4/80 (~45%), while the percentages of these two types of cells in S1pr2^+^ or S1pr3^+^ cells are much lower (Figure 4D–I). Moreover, in both CD31^+^ and F4/80^+^ cells, S1pr1 is the most common type of S1P receptor (~50% and ~25%, respectively), while the presence of S1pr2 or S1pr3 is much rarer (Figure 4E–J). Together, these data indicate that endothelial cells and resident macrophages in mouse SMGs are the major sources of extracellular S1P and the major cells responsive to the pro-regenerative S1pr1 signaling.

### 3.5. S1P Protects Salivary Gland Macrophages and Endothelial Cells from Irradiation Damage

**Figure 5:**
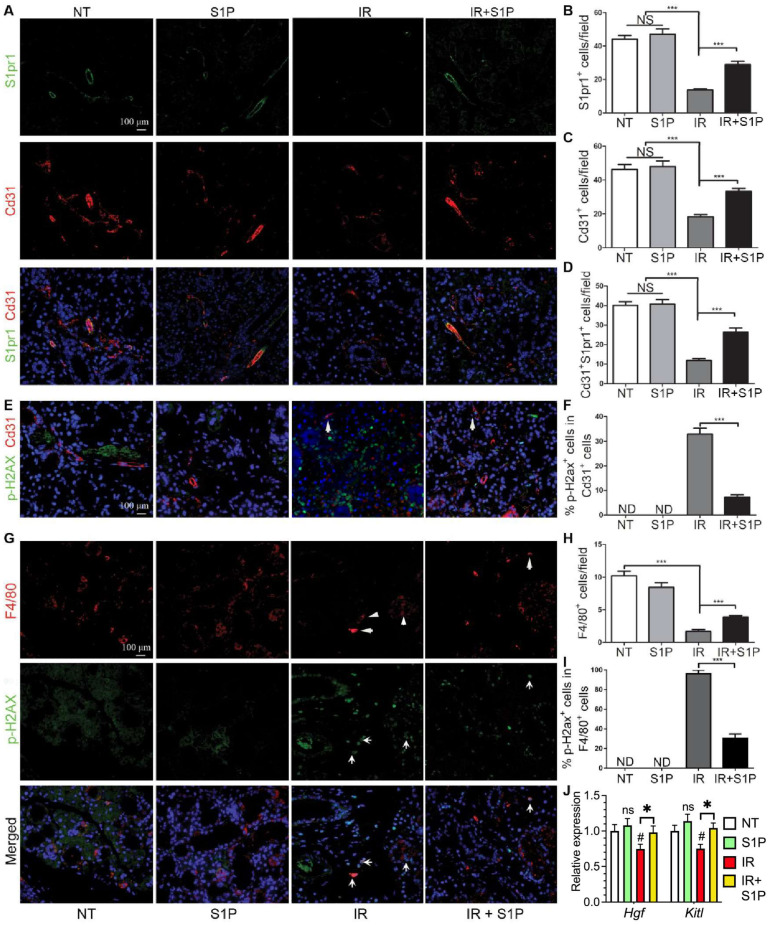
**Effects of S1P on endothelial cells and macrophages in radiated salivary glands.** (**A**–**D**) Expression of S1pr1 and Cd31 in SMGs collected at 7 days after IR were examined with double immunofluorescence staining and quantified. (**E**,**F**) Levels of p-H2ax in Cd31^+^ endothelial cells in SMGs collected at 7 days after IR were examined with double immunofluorescence staining and quantified. (**G**–**I**) Expression of F4/80 and levels of p-H2ax in F4/80^+^ macrophages in SMGs collected at 7 days after IR were examined with double immunofluorescence staining and quantified. (**J**) mRNA levels of macrophage-enriched Hgf and endothelia-enriched Kitl in SMGs collected at 7 days after IR were examined with RT-qPCR. Quantified data are shown as mean ± SD, N = 3, NS: not significant vs. NT, ND: not detected, ^#^: *p* < 0.05 vs. NT, *: *p* < 0.05, ***: *p* < 0.001. Quantitative data were analyzed using one-way ANOVA with Tukey’s multiple comparisons.

SMG-resident macrophages and endothelial cells are essential for maintaining salivary function, are severely damaged by IR [5,7], and highly express pro-regenerative S1pr1 (Figure 4). Therefore, we examined the effects of IR with or without S1P treatment on S1pr1^+^ cells, endothelial cells, and macrophages by the immunofluorescence staining of SMGs collected 7 days after IR or S1P treatment. The numbers of S1pr1^+^ cells, Cd31^+^ endothelial cells, and S1pr1^+^Cd31^+^ cells were not significantly affected by S1P treatment alone, greatly decreased by IR, as reported previously [5,43], and preserved by S1P treatment before IR (Figure 5A–D). In the NT and S1P groups, no Cd31^+^ endothelial cells were positive for the DSB marker p-H2ax, whereas many remaining Cd31^+^ endothelial cells in the IR group became p-H2ax^+^, which was also significantly decreased by S1P treatment before IR (Figure 5E,F and Figure S8). Similarly, the number of F4/80^+^ macrophages was not significantly affected by S1P treatment alone, was greatly decreased by IR, as we recently reported [7], and was preserved by S1P treatment before IR (Figure 5G,H). In the NT and S1P groups, no F4/80^+^ macrophages were p-H2ax^+^, whereas most F4/80^+^ macrophages in the IR group became p-H2ax^+^, which was also significantly decreased by S1P treatment before IR (Figure 5G,I). Consistently, the mRNA levels of rMφ-enriched *Hgf* [7] and endothelia-enriched *Kitl* in SMGs collected 7 days after IR significantly decreased compared with the NT group, which was reversed by S1P treatment before IR (Figure 5J). These data indicate that SMG endothelial cells and resident macrophages are key targets for the radioprotective effects of S1P, while the preservation of epithelial homeostatic factors produced by macrophages and endothelial cells contributes to the radio-protective effect of S1P on SMG function.

### 3.6. S1P Activates S1pr1/Akt/eNOS Pathway in Radiated Endothelial Cells and Salivary Glands

**Figure 6:**
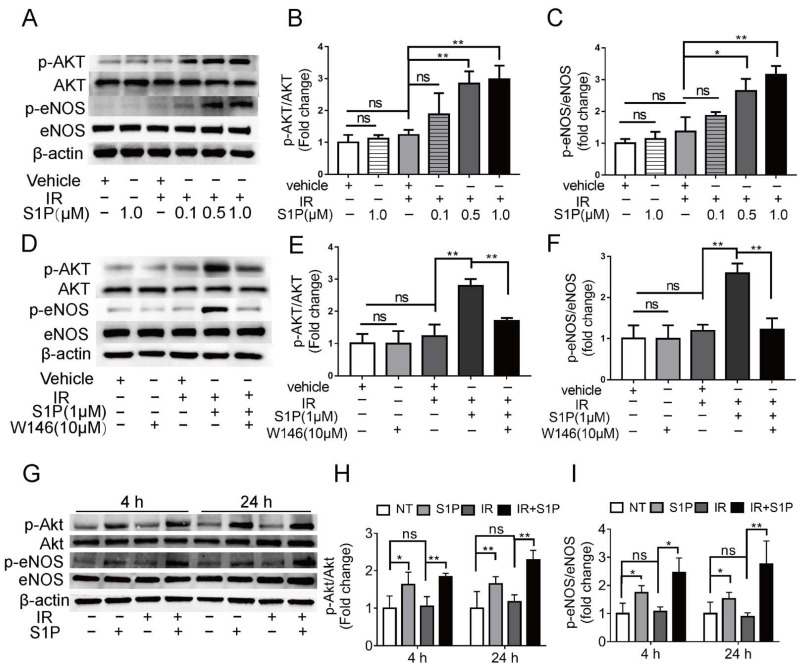
**Effects of S1P on S1pr1/Akt/eNOS pathway in radiated endothelial cells and SMGs.** (**A**–**F**) HUVECs were treated with IR, IR + S1P, S1PR1 inhibitor W146, or IR + S1P + W146 for 4 h. Activation of AKT/eNOS pathway in these cells was examined with Western blot for phosphorylated and total AKT and eNOS. (**G**–**I**) In mouse SMGs collected 4 or 24 h after IR and/or S1P treatment, activation of Akt/eNOS pathway was examined with Western blot. Quantified data are shown as mean ± SD, N = 3, ns: not significant, *: *p* < 0.05, **: *p* < 0.01. Quantitative data were analyzed using one-way ANOVA with Tukey’s multiple comparisons.

To explore the underlying mechanisms of S1P in preserving endothelial cells after IR, we treated human umbilical vein endothelial cells (HUVECs) with 0.1, 0.5, or 1 μM S1P at 30 min before receiving 10 Gy IR. At 24 h after IR, immunofluorescent staining and Western blot analyses indicated that IR significantly increased ROS levels and NOX4 expression and decreased NRF2 expression, which were both inhibited by S1P treatment in a dose-dependent manner (Supplementary Figure S6A–E). These results demonstrated that S1P protected HUVECs from IR damage through anti-oxidative effects as found in mouse SMGs. In endothelial cells from other organs, S1P activates Akt and the endothelial isoform of nitric oxide synthase (eNOS) signaling pathways [44], while both Akt signaling and nitric oxide can enhance Nrf2-dependent antioxidant functions [45,46]. In HUVECs examined 4 h after IR, phosphorylation levels of AKT and eNOS were not significantly affected by PET vehicle, S1P alone, or IR alone but upregulated by S1P treatment before IR in a dose-dependent manner (Figure 6A–C). The effect of S1P itself on Akt/eNOS pathway in cultured HUVECs was reported transient (5–20 min) [47], and we did not detect a significant effect of S1P alone treatment at 4 h. Therefore, the prolonged activation of Akt/eNos pathway by S1P plus IR might be due to the increased production of endogenous S1P from ceramides induced by radiation [32,33]. S1PR1 is the major S1P receptor in endothelial cells, including SMGs (Figure 4). To determine whether the protective effect of S1P on endothelial cells was mediated by S1R1 signaling, HUVECs were treated with a selective S1PR1 inhibitor, W146 [48], either alone or in combination with 1 μM S1P and IR. W146 treatment did not significantly affect the phosphorylation levels of Akt and eNOS, but upregulation of these indices was significantly inhibited by S1P in irradiated HUVECs (Figure 6D–F). Consistently, in HUVECs, W146 treatment did not significantly affect the protein levels of NOX4 and Nrf2 but abolished the inhibitory effects of S1P on IR-induced NOX4 upregulation and Nrf2 downregulation (Supplementary Figure S9F–H). The restoration of Nrf2 expression after IR by S1P was also blocked by the inhibition of Akt signaling with MK2206 (Supplementary Figure S10). These results showed that S1P pretreatment protected HUVECs from IR damage through the S1PR1/AKT/eNOS pathway. In mouse SMGs collected at 4 and 24 h after IR or S1P treatment, levels of phosphorylated Akt and eNOS were not significantly affected by IR alone but increased by S1P treatment with or without IR (Figure 6G–I). The in vivo effects of S1P alone treatment on Akt/eNOS pathway were much more persistent than that on cultured endothelial cells, which is likely due to the involvements of other S1P-responsive cells such as macrophages. These data collectively suggest that S1P protects SMGs from IR injury through the S1pr1/Akt/eNOS pathway, as observed in cultured endothelial cells.

### 3.7. Intra-SMG Injected S1P Does Not Promote Growth or Radio-Resistance of Head and Neck Cancer

Since the ultimate goal of our research was to preserve saliva secretion in patients with head and neck cancer, it is necessary to determine whether S1P shows any pro-tumor potential. First, intra-SMG S1P injection at the highest dose (0.2 mg/kg) did not significantly affect the plasma S1P level (Supplementary Figure S11). Second, in cultured CAL27 human head and neck cancer cells [12] with or without 10 Gy radiation, 1 μM S1P treatment did not significantly affect the proliferation of CAL27 cells (Supplementary Figure S12A). Lastly, we examined whether intra-SMG administration of S1P showed any pro-tumor effect in nude mice carrying subcutaneous CAL27 tumors at their flanks. These mice were treated with 0.2 mg/kg intra-SMG S1P, 15 Gy local irradiation of tumors, or S1P with irradiation. Observation for 15 days after treatment indicated that S1P did not significantly affect the tumor volumes or endpoint tumor weights, or the responses to local radiation therapy (Supplementary Figure S12B–D). Consistently, intra-SMG S1P treatment did not significantly affect the levels of the endothelial marker CD31 and the proliferation marker Ki67 in tumors with or without local IR (Supplementary Figure S9E–G). These data indicate that intra-SMG injection of S1P did not promote the growth or radioresistance of tumors outside the SMGs.

## 4. Discussion

S1P, a natural bioactive lipid, is an essential regulator of many physiological and pathogenic processes in multiple vertebrate organs, and S1P metabolic enzymes, transporters, and receptors are promising therapeutic targets for various diseases [9]. We found that S1P pretreatment preserved salivary function after IR in mice by decreasing IR-induced oxidative stress and the consequent DNA damage, apoptosis, and cellular senescence. Our scRNA-seq data indicated that salivary gland endothelial cells and resident macrophages are major sources of endogenous S1P and highly express the pro-regenerative S1P receptor S1pr1. These two types of cells are essential early targets of IR damage [5,6,7], and S1P pretreatment effectively protects these cells and SMGs from IR damage.

Saliva secretion is dependent upon the blood supply to salivary glands [49,50], whereas the radiation-induced injury of microvascular endothelial cells led to diminished nourishment and, thus, to long-term dysfunction of salivary epithelial cells [5,6]. SMG endothelial cells also produce growth factors that regulate the homeostasis of other cells, such as Csf1 for resident macrophages [7] and Kitl for Kit^+^ epithelial progenitors. Consistently, *Kitl* expression was significantly decreased by radiation but restored by S1P pretreatment. Therefore, the increased survival of endothelial cells by S1P pretreatment preserved the overall function of SMGs through the restoration of blood supply, nourishment, and homeostatic paracrine factors. Besides endothelial cells, peri-endothelial cells, including smooth muscle cells, pericytes, and their CD31/Pecam1^−^CD34^+^ progenitors, are also essential for vascular morphogenesis and stabilization in salivary glands after irradiation [35]. Although much rarer than endothelial cells, our data did find *S1pr1* expression in some peri-endothelial progenitors and pericytes. S1P has been reported to induce the differentiation of smooth muscle cells from progenitors [51,52] and protect pericytes from the inflammatory damage [53], which likely also contribute to the radio-protective effects of S1P on salivary glands.

We have reported that salivary gland resident macrophages are the major source of Hgf that regulates the homeostasis of Met^+^ epithelial progenitors, whereas radiation rapidly and persistently decreased the number of resident macrophages and Hgf level [7], which were both prevented by S1P pretreatment. Moreover, as in other tissues, resident macrophages are capable of clearing dead and senescent cells without causing severe inflammation through efferocytosis [54], which is also essential for the preservation of salivary gland function after irradiation [18]. Therefore, the increased survival of resident macrophages by S1P pretreatment preserved the overall function of SMGs through both efferocytosis and homeostatic paracrine interactions with other cells.

We confirmed that the radioprotective effect of S1P on cultured endothelial cells was mediated by S1PR1 and downstream AKT and eNOS signaling pathways [55]. In mouse SMGs, S1P also enhances the activities of Akt/eNOS pathways that can upregulate Nrf2-medicated anti-oxidative responses [45,46], suggesting that these pathways mediate the in vivo radioprotective effect of S1P. Other mechanisms may also contribute to the upregulation of Nrf2 by S1PR1 signaling. For instance, S1PR1 signaling cross-talks with the signaling pathway of lysophosphatidic acid (LPA), another bioactive lysophospholipid activating GPCR and abundant in saliva [56,57], while LPA signaling stabilizes Nrf2 through PI3K/Akt signaling [58]. We will explore this potential mechanism in our future work.

Many SMG resident macrophages are associated with blood vessels [59] and interact with endothelial cells through homeostatic paracrine/juxtacrine factors, such as Csf1 and C1q [7]. It has been reported that after myocardial infarction, endothelial S1pr1 signaling promotes tissue repair by stimulating tissue-resident (reparative) macrophage proliferation [60]. Therefore, the radioprotective effects of S1P on endothelial cells and resident macrophages could be mediated by both the direct activation of S1pr1 signaling in these two types of cells and the consequent enhancement of homeostatic interactions between these cells. Besides S1pr1, whether other S1PRs are involved in the maintenance of salivary glands is worth further exploring.

IR immediately increases ceramide levels through the hydrolysis of sphingomyelin by Smpd1 and Smpd2 [32,33], which enhances apoptosis and leads to IR damage in multiple organs, including the salivary glands [31]. Both Smpd1 and Smpd2 are abundant in SMG endothelial cells and may contribute to the high sensitivity of endothelial cells to IR, as reported in other organs [61]. However, ceramidases and sphingosine kinases can metabolize ceramides to S1P, and extracellular S1P acts as a radioprotectant in other organs [62]. We found that SMG endothelial cells and resident macrophages expressed these S1P-producing enzymes and S1P-transporters at high levels, suggesting that the rapid decrease in these cells after IR might trigger a vicious circle of imbalance between pro-apoptotic ceramides and anti-apoptotic S1P, which enhances the apoptosis of more SMG cells. Conversely, it is plausible that intra-SMG S1P pretreatment decreased IR-induced apoptosis by preserving these two major types of S1P-producing cells and the consequent balance between ceramides and S1P.

In multiple types of cancers, including some head and neck cancer, S1P signaling is considered pro-tumoral as it promotes cell proliferation, survival, migration, inflammation, and angiogenesis [9]. However, in some solid tumors, such as pancreatic cancer, increasing plasma S1P levels is a promising strategy to decrease tumor hypoxia through vascular normalization and enhance therapeutic efficacy [63]. To avoid the pro-tumor potential of S1P, we injected S1P locally into the submandibular glands. In our mouse model of head and neck cancer, intra-SMG S1P dosage did not promote cancer growth or resistance to radiation therapy. These data suggest that local S1P appears safe for preserving saliva secretion in patients with head and neck cancer treated with radiotherapy.

## 5. Conclusions

Our results demonstrate that S1P pretreatment mitigates salivary gland dysfunction after IR by preserving endothelial cells and resident macrophages, likely through the S1pr1/Akt/Nrf2 pathways. These data indicate that S1P signaling pathway is a promising target for preserving saliva sections after radiation therapy for head and neck cancers. In addition, our data also suggest that S1P signaling regulates the homeostasis of salivary endothelial cells and resident macrophages, which is essential for salivary gland function.

## Data Availability

The scRNA-seq dataset has been deposited to Gene Expression Omnibus with the record number GSE204843. The data presented in this study is contained in the article and Supplementary Materials.

## Supplementary Materials

The following supporting information can be downloaded at: https://www.mdpi.com/article/10.3390/antiox11102050/s1, Figure S1: The setup of irradiation; Figure S2: Proof-of-principle of percutaneous intra-SMG injections; Figure S3: Changes of salivary flow rate over 90 days after IR with or without S1P pretreatment; Figure S4: Magnified images of TUNEL staining shown in Figure 2A; Figure S5: Magnified images of p-H2ax staining shown in Figure 3A; Figure S6: Further gene expression analyses of the Endo cluster shown in Figure 4A; Figure S7: The validation of gene expression patterns with RT-qPCR of FACS sorted SMG cells; Figure S8: Single channels of p-H2ax and CD31 signals without DAPI for merged images in Figure 5E; Figure S9: S1P protected HUVECs from radiation-induced oxidative stress through S1PR1-mediated upregulation of Nrf2; Figure S10: The restoration of Nrf2 expression after IR by S1P was blocked by AKT inhibition; Figure S11: Plasma S1P levels after intra-SMG S1P injection; Figure S12: Effects of intra-SMG injected S1P on established head and neck cancer. Reference [64] is cited in the Supplementary Materials.
