# Supplementary material for: Sphingosine-1-Phosphate Alleviates Irradiation Induced Salivary Gland Hypofunction through Preserving Endothelial Cells and Resident Macrophages
Source: Antioxidants (Basel). 2022 Oct 18;11(10):2050. doi: 10.3390/antiox11102050 (PMC9598384; doi:10.3390/antiox11102050)
Supplement: Supplementary file 1 [file antioxidants-11-02050-s001.zip › antioxidants-1869453-supplementary.pdf]

Supplementary Data

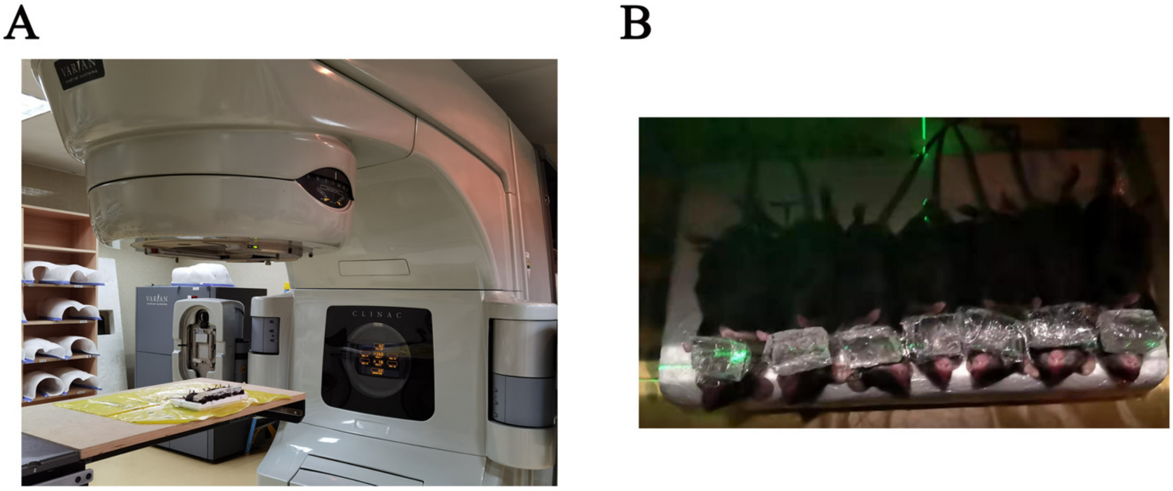

Figure S1. The irradiation of mice. (**A**,**B**) The irradiation machine and the radiation filed of mice. Mice were anesthetized and put at supine position. The irradiated area was located at submandibular area. The IR therapy plan was 6 mV of photon energy at 3 Gy/min by Elekta Synergy accelerator (Trilogy, Varian, CA, United States). Animals received a 15 Gy single dose IR.

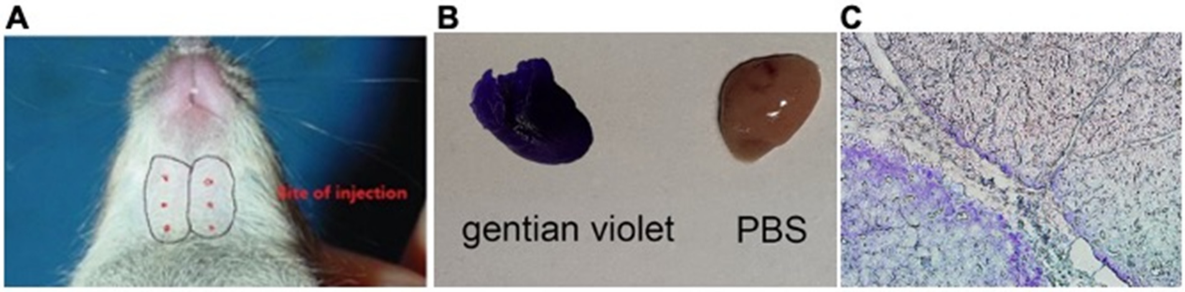

Figure S2. Proof-of-principle of percutaneous intra-SMG injections. A. For percutaneous intra-SMG injections, 31-gauge insulin syringes were used. After disinfection with 70% isopropyl alcohol, the neck skin of mice was pulled up to allow the needle penetrating the skin at an angel <30 degree for 3-5 mm to reach the tough SMG capsule with increased resistance. The needle was further pushed to penetrate SMG capsule at an angel <45 degree, and then agents in syringes were injected. Injection was repeated at 3 different sites per SMG to ensure the homogeneous distribution of injected agents in SMGs. B-C. Gentian violet dye or PBS was injected into the right SMG using above method. The whole mount images of both SMGs and a section of Gentian violet injected SMG were shown as the proof-of-principle of percutaneous intra-SMG injections.

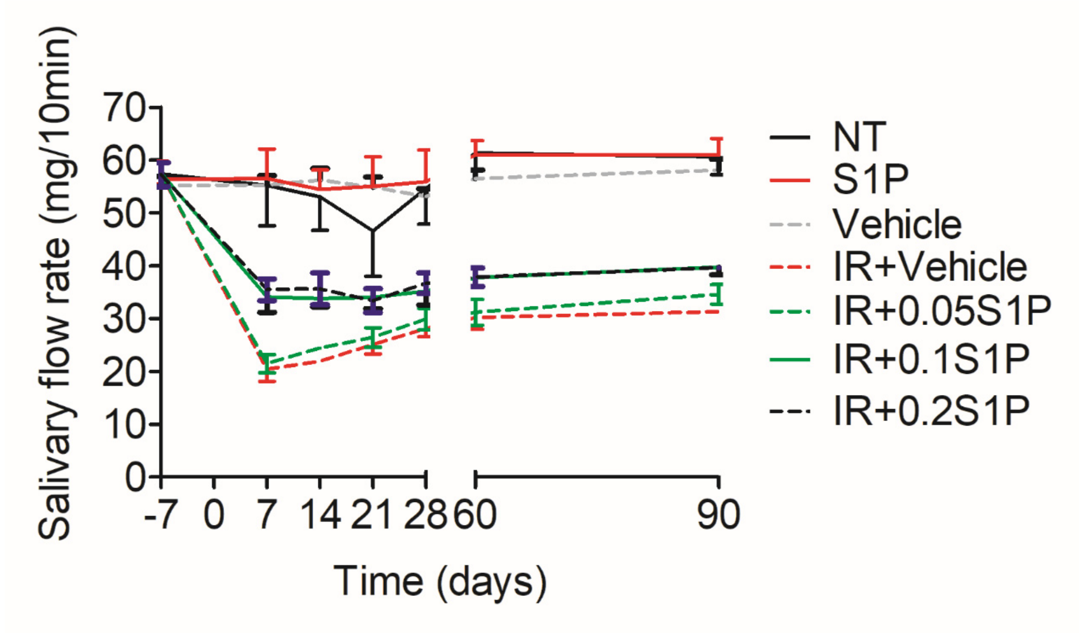

Figure S3. Changes of salivary flow rate over 90 days after IR with or without S1P pretreatment. From 7 day before to 90 days after IR with or without S1P pretreatment, mice were anaesthetized to collect the whole saliva for 10 mins. The salivary flow rate maintained at around 60 mg/10 min in NT group, S1P group and Vehicle group at all time points, greatly decreased from 7 days after IR and maintained at low levels. S1P pretreatment at 0.1 or 0.2 mg/kg but not at 0.05 mg/kg significantly improved salivary flow rate from 7 to 90 days after IR.

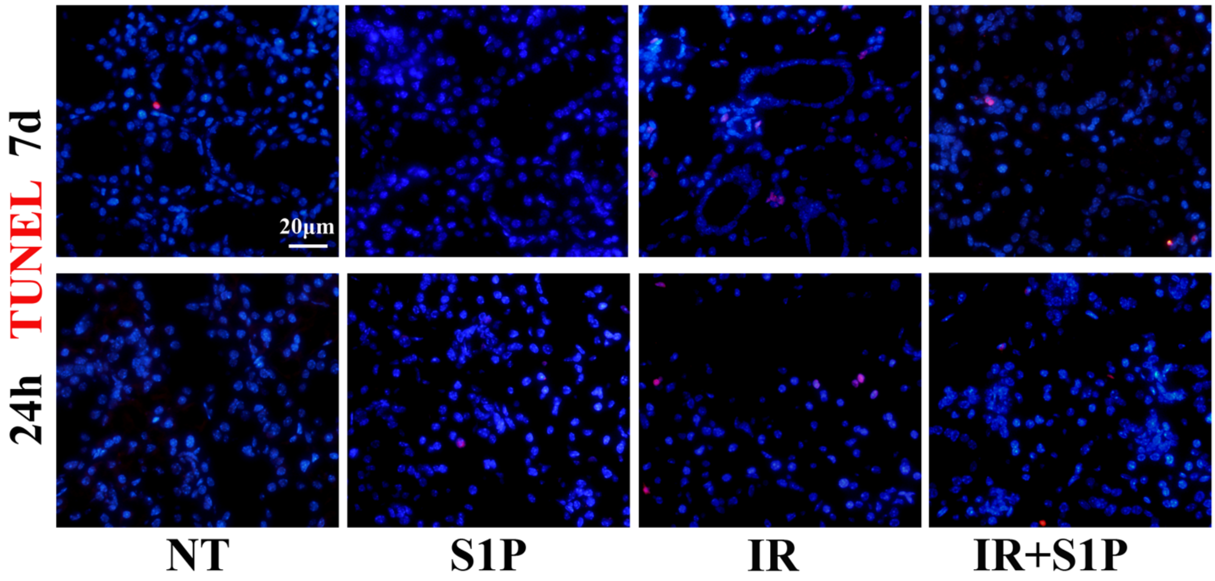

Figure S4. Magnified images of TUNEL staining shown in Figure 2A.

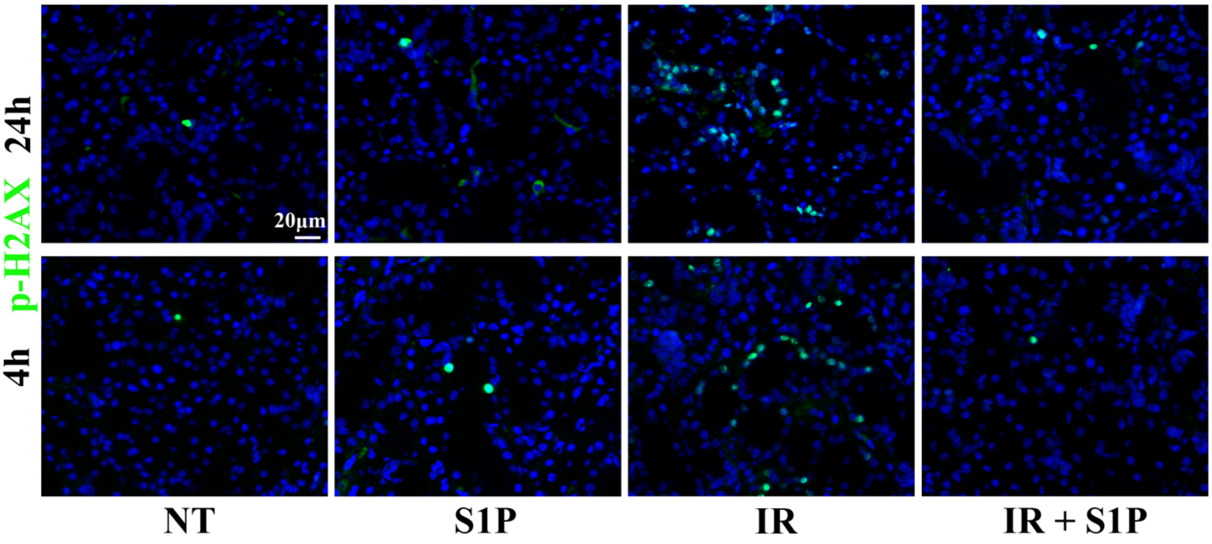

Figure S5. Magnified images of p-H2ax staining shown in Figure 3A.

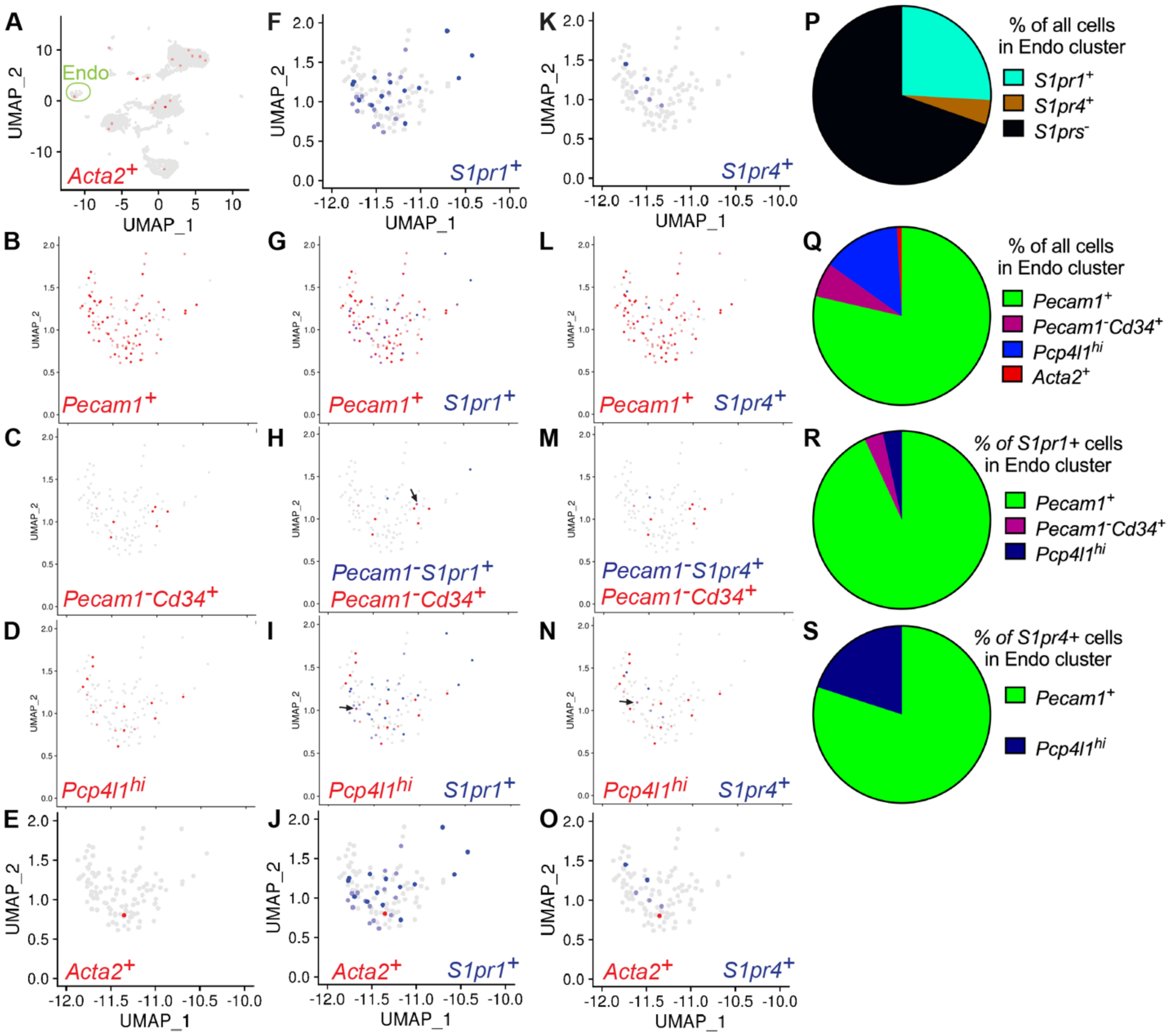

Figure S6. Further gene expression analyses of the Endo cluster shown in Figure 4A. (A) The expression pattern of smooth muscle cell marker *Acta2* were shown in a feature plot of all clusters of scRNA-seq data of nontreated SMGs. Endo: endothelial and peri-endothelial cells. (B-O) Expression patterns of endothelial/peri-endothelial marker genes and S1P receptors were shown in feature plots of the Endo cluster. Cells expressing both genes in Panels H-I and N are labeled with arrows as purple dots. (P-S) Percentages of cells expressing endothelial/peri-endothelial marker genes and/or S1P receptors in the Endo cluster were shown as pie charts.

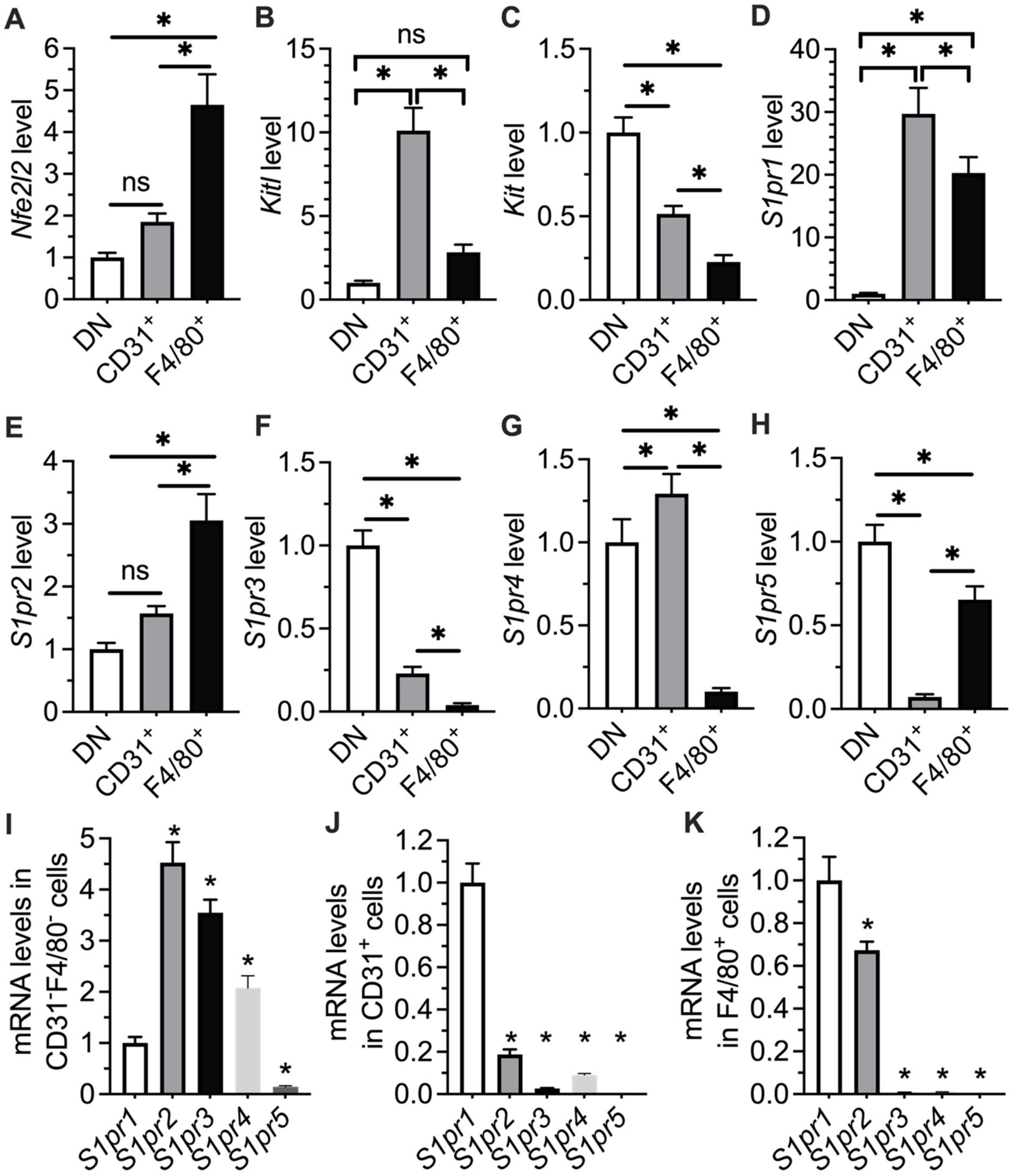

Figure S7. The mRNA expression patterns of Nfe2l2, Kitl, Kit, and S1prs were validated with RT-qPCR of FACS sorted SMG cells. SMGs were collected from non-treated 8-10 weeks old male C57BL/6 mice and sorted with FACS for CD31^{+}, F4/80^{+}, and CD31^{-}F4/80^{-} double negative (DN) cells. These cells were analyzed with RT-qPCR for the mRNA expression levels of Nfe2l2, Kitl, Kit, and S1prs. N = 3. mRNA levels were normalized with the reference gene *Gapdh*. (A-H) The expression of each gene was shown as relative levels compared to normalized levels of corresponding genes in

double negative cells. *: $p < 0.05$, ns: not significant. (I-K) The expression of 5 S1PRs was shown as relative levels compared to normalized levels of *S1pr1* in $CD31^+$, $F4/80^+$, and $CD31^-F4/80^-$ (DN) cells respectively. *: $p < 0.05$ vs. *S1pr1*. Data are expressed as mean ± SD and analyzed using One-way ANOVA with Tukey's multiple comparisons.

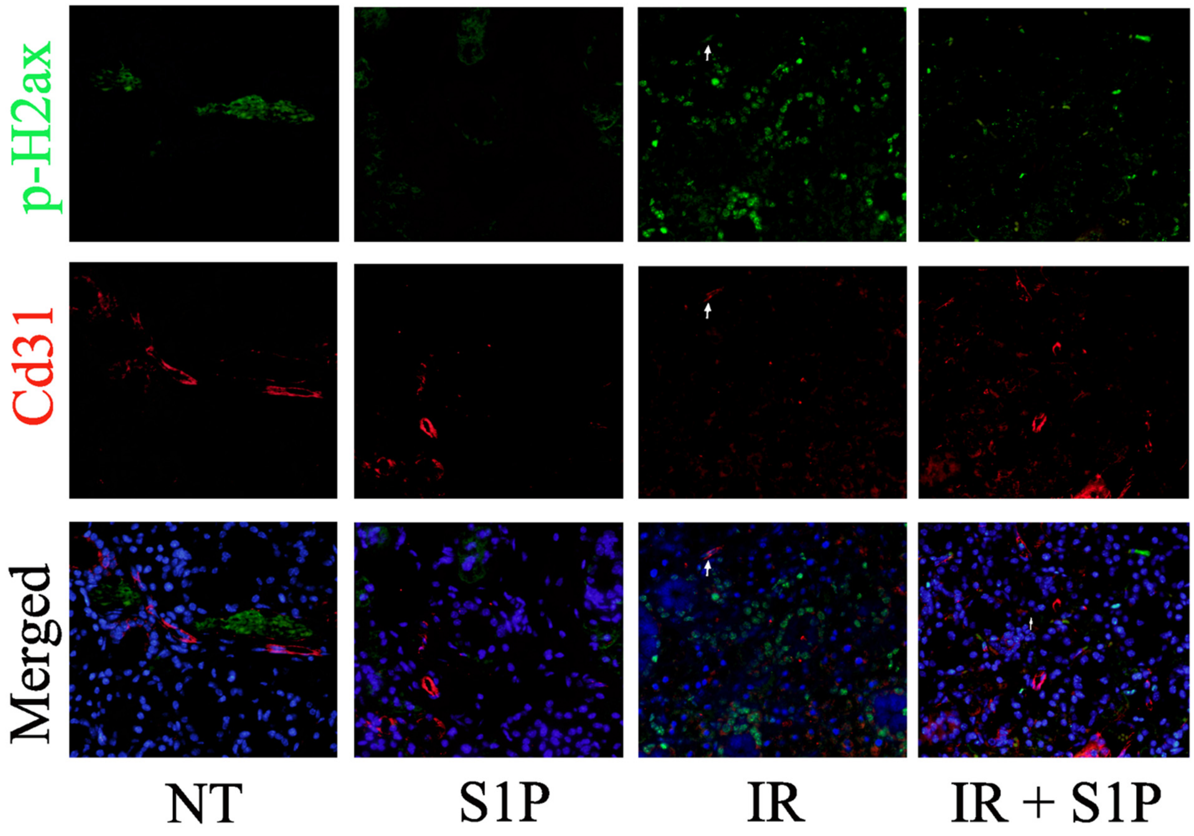

Figure S8. Single channels of p-H2ax and CD31 signals without DAPI for merged images in Figure 5E. p-H2ax staining (green) and CD31 staining (red) were imaged separately and then merged with DAPI images.

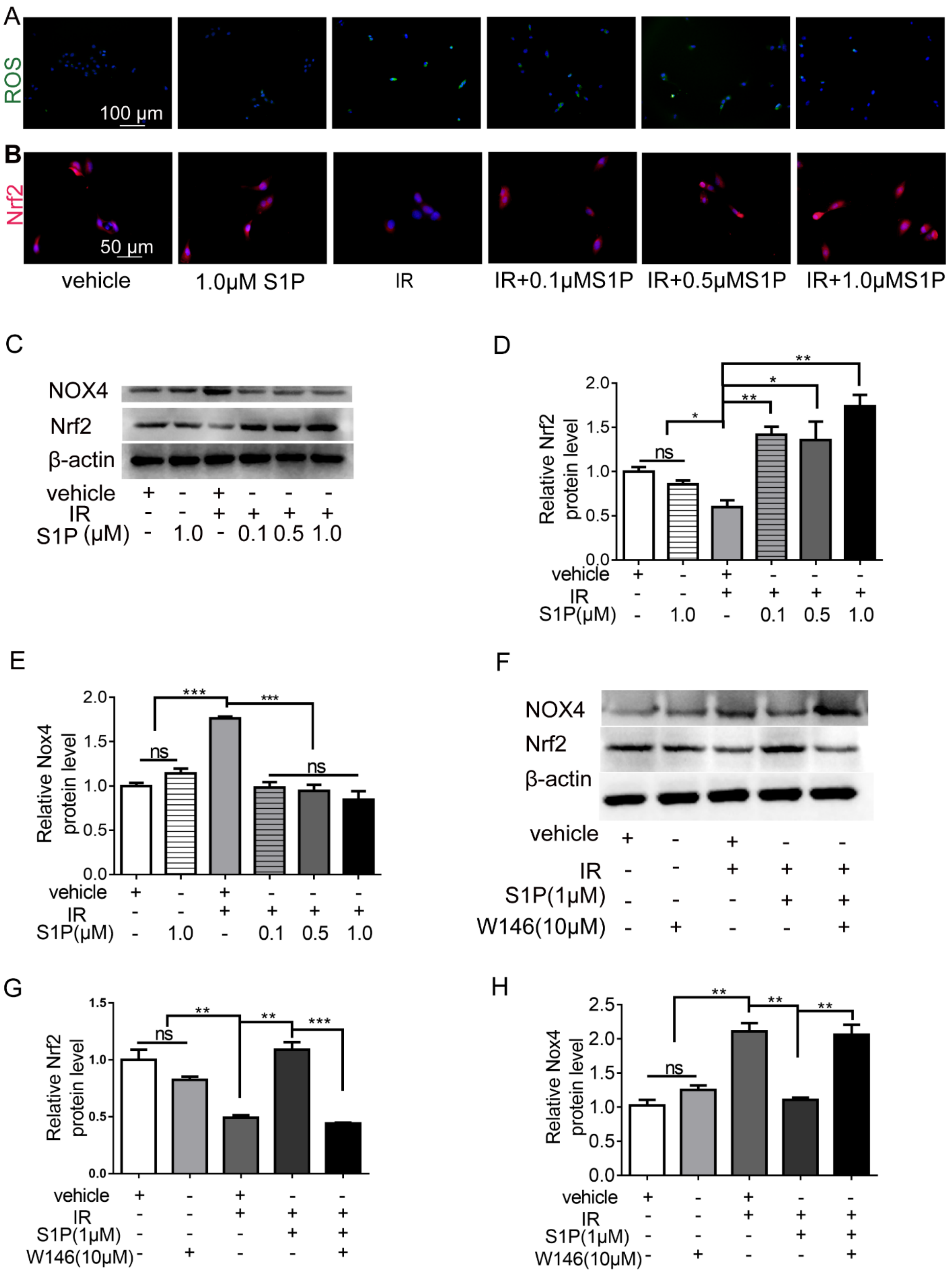
A
ROS
100 μm
B
Nrf2
50 μm
vehicle
1.0μM S1P
IR
IR+0.1μMS1P
IR+0.5μMS1P
IR+1.0μMS1P
C
NOX4
Nrf2
β-actin
vehicle + - + - - -
IR - - + + + +
S1P (μM) - 1.0 - 0.1 0.5 1.0
D
Relative Nrf2 protein level
2.0
1.5
1.0
0.5
0.0
ns
*
**
*
**
vehicle + - + - - -
IR - - + + + +
S1P(μM) - 1.0 - 0.1 0.5 1.0
E
Relative Nox4 protein level
2.0
1.5
1.0
0.5
0.0
***
***
ns
ns
vehicle + - + - - -
IR - - + + + +
S1P(μM) - 1.0 - 0.1 0.5 1.0
F
NOX4
Nrf2
β-actin
vehicle + - + - -
IR - - + + +
S1P(1μM) - - - + +
W146(10μM) - + - - +
G
Relative Nrf2 protein level
1.5
1.0
0.5
0.0
ns
**
**
***
vehicle + - + - -
IR - - + + +
S1P(1μM) - - - + +
W146(10μM) - + - - +
H
Relative Nox4 protein level
2.5
2.0
1.5
1.0
0.5
0.0
ns
**
**
**
vehicle + - + - -
IR - - + + +
S1P(1μM) - - - + +
W146(10μM) - + - - +

Figure S9. S1P protected HUVECs from radiation-induced oxidative stress through S1pr1-mediated upregulation of Nrf2. (A) The ROS levels in HUVECs treated with irradiation and/or S1P were detected at 24 hours after irradiation with a commercial kit. (B) The level and subcellular location of Nrf2 in HUVECs were detected at 24 hours after irradiation with Immunofluorescence staining (IF). (C-H) Levels of Nrf2 and NOX4 proteins in HUVECs treated with S1P and/or S1pr1 specific antagonist W146 were detected at 24 hours after irradiation with Western blot analysis and quantified by Image J. Data are shown as the mean ± SD, N = 3, *p < 0.05, **p< 0.01, ***p < 0.001.

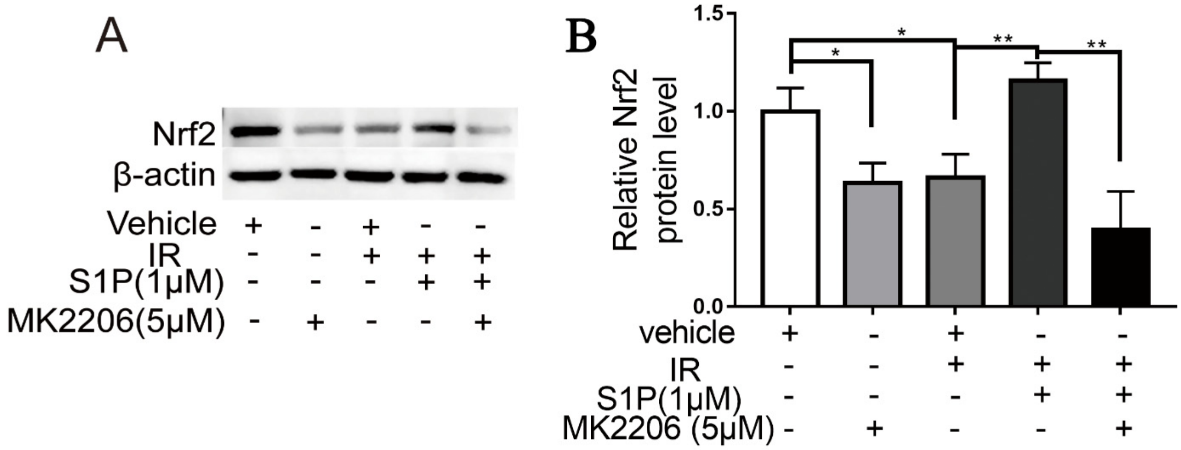

Figure S10. The restoration of Nrf2 expression after IR by S1P was blocked by AKT inhibition. HUVECs were treated with 5 μm AKT inhibitor MK2206 (Konieczny et al. 2022) at 3 hours prior to IR and collected at 24 hours after IR. Nrf2 protein was detected with western blot (A) and quantified using Image J (B).

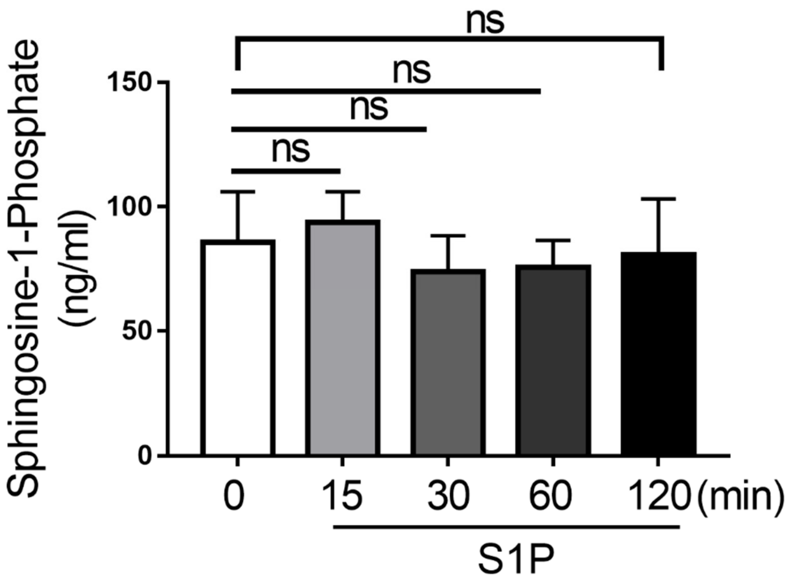

Figure S11. Plasma S1P concentration after intra-SMG S1P injection. We detected the plasma S1P concentration in mice at 0 min to 120 mins after intra-SMG injection of 0.2 mg/kg S1P. Our results showed that the plasma S1P concentration was not significantly affected by intra-SMG S1P injection.

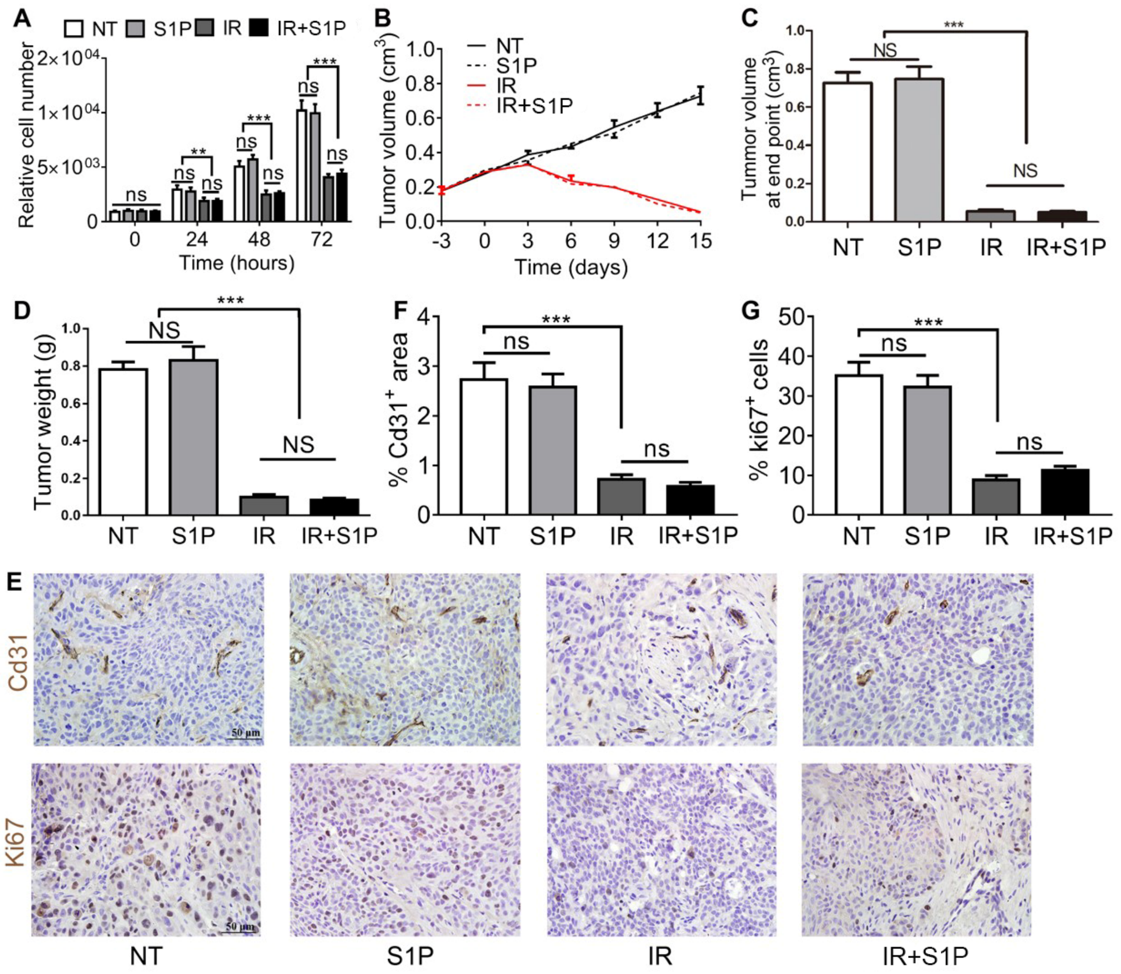

Figure S12. Effects of intra-SMG injected S1P on established head and neck cancer. (A) CAL27 cells were treated with S1P, IR, or IR+S1P, and cell numbers were counted at 24, 48 or 72 hours after treatment. (B-D) Nude mice carrying established subcutaneous CAL27 tumor were treated with intra-SMG S1P, IR on tumor, or IR+S1P. Tumor volumes were measured every 3 days, and tumors were collected and weighed at 15 days after treatment. (E-G) Tumor sections were examined with IHC for endothelial marker Cd31 and proliferation marker Ki67. Cd31+ areas and Ki67+ cells were quantified from 3 representative fields per sample. Quantified Data are showed as mean ± SD, N = 6, ns: not significant, ***: $p < 0.001$.

Reference

1. Konieczny P, Xing Y, Sidhu I, Subudhi I, Mansfield KP, Hsieh B, Biancur DE, Larsen SB, Cammer M, Li D et al. 2022. Interleukin-17 governs hypoxic adaptation of injured epithelium. Science. 377(6602):eabg9302.
